# Beyond energy: how TCA cycle-derived metabolites regulate gene expression and inflammation in the nucleus

**DOI:** 10.1186/s12950-025-00461-x

**Published:** 2025-09-26

**Authors:** Jaiya Randhawa, Eva M. Pålsson-McDermott

**Affiliations:** School of Biochemistry and Immunology, Trinity Biomedical Science Institute, Trinity College Dublin, Dublin 2, Ireland

**Keywords:** Immunometabolism, TCA cycle, Inflammation, Epigenetics, Metabolites

## Abstract

Immune cells can rewire their metabolism in response to various stimuli. Crosstalk between the nucleus and mitochondria allows for tight regulation of this metabolic reprogramming. Research has emerged showing several TCA cycle-derived metabolites exhibiting moonlighting functions in the nucleus, modulating chromatin modifications in order to control inflammation. These TCA cycle-derived metabolites include acetyl-CoA, α-ketoglutarate, succinate, fumarate, itaconate, and succinyl-CoA which can modify DNA or histone to drive or inhibit gene expression. In this review, we look at the mechanisms of TCA cycle metabolites’ non-canonical functions in the nucleus in the context of inflammation. In addition, we discuss the known and possible links between these metabolites’ nuclear moonlighting functions and the pathogenesis of diseases, including inflammatory diseases and cancers.

**Supplementary Information:**

The online version contains supplementary material available at 10.1186/s12950-025-00461-x.

## Background

Immunometabolism is the study of how metabolic pathways and metabolites impact immune effector cells’ function, activation and response [1]. More specifically, the field of immunometabolism explores how various metabolic pathways including glycolysis, the tricarboxylic acid (TCA) cycle, the pentose phosphate pathway, amino acid metabolism, and fatty acid oxidation and synthesis, convert extracellular nutrients into intracellular metabolites, thereby altering and regulating the effector functions of a given immune cell in the process [2, 3].

Upon activation the metabolism of an immune cell changes and adapts to better execute an appropriate response. First and foremost, the alteration in cellular metabolism acquires the molecules needed for energy and cell division [4]. Macrophages exhibit remarkable plasticity and can adopt a spectrum of functional phenotypes in response to microenvironmental signals. While the M1/M2 nomenclature has historically been used to describe classically activated, pro-inflammatory (M1-like) and alternatively activated, anti-inflammatory or tissue-repairing (M2-like) states, this dichotomy is now recognized as an oversimplification. Contemporary understanding acknowledges a continuum of macrophage activation states, shaped by complex and overlapping signaling pathways. Nevertheless, the M1/M2 framework can still be useful as a heuristic for describing dominant functional polarizations under specific experimental or pathological conditions [5]. For example, when presented with an inflammatory stimulus, macrophages polarize towards an inflammatory phenotype which requires a metabolic shift towards aerobic glycolysis, also known as Warburg metabolism [6, 7]. Under anti-inflammatory conditions, many immune cells, such as macrophages, favour oxidative phosphorylation metabolism, which can yield 32 adenosine triphosphate (ATP) molecules [4] per glucose molecule metabolised. Although aerobic glycolysis is favoured during inflammation, the TCA cycle is additionally altered, causing an accumulation of specific metabolites. Several of these metabolites, including succinate, fumarate and itaconate have been shown to regulate the immune responses [8, 9].

**Fig. 1:**
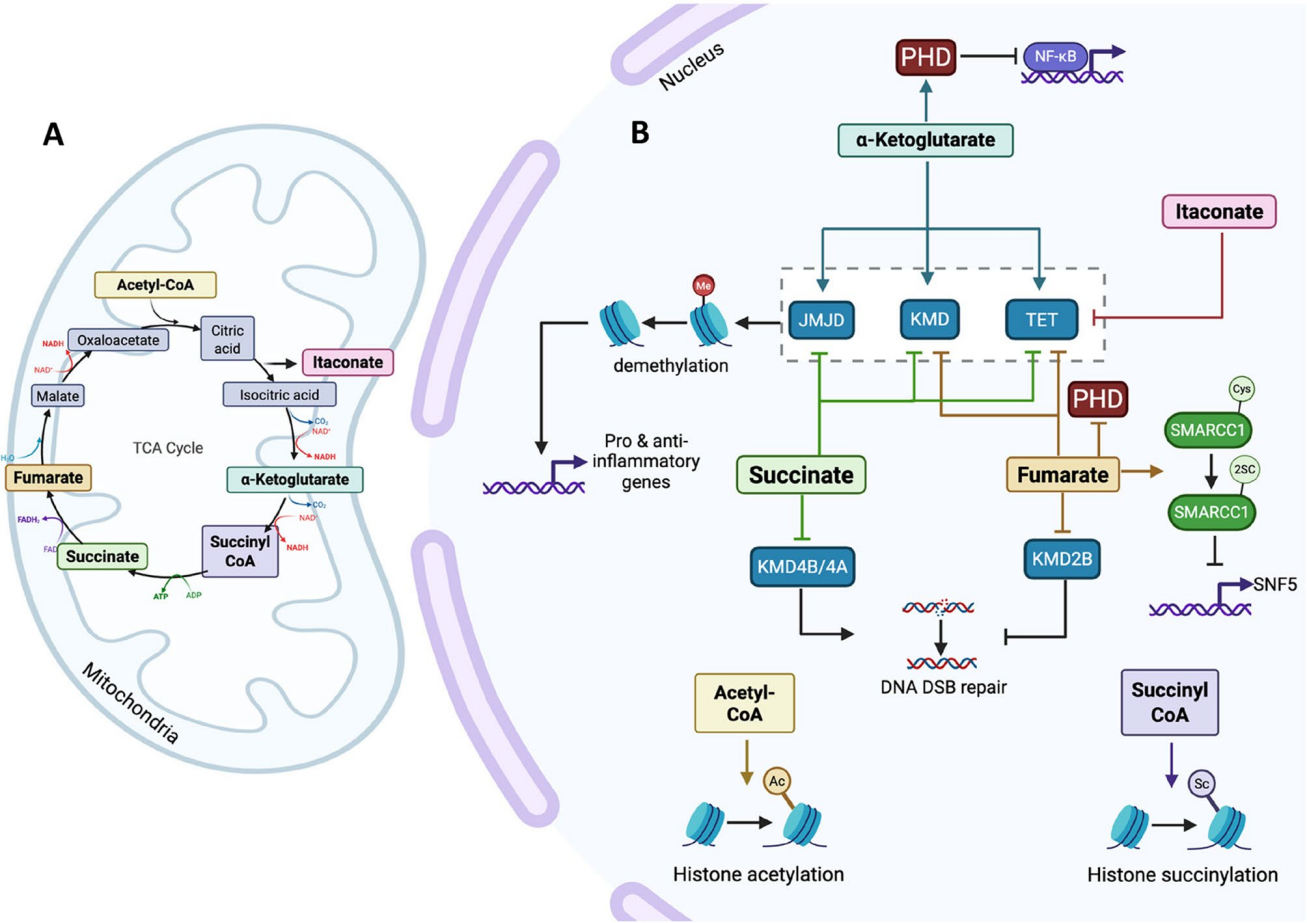
TCA-cycle metabolites moonlight in the nucleus, altering gene expression. **(A)** Mitochondrial/TCA cycle functions. Pyruvate generally derived from glycolysis, is converted to Acetyl-CoA, which combines with the metabolite oxaloacetate in the presence of citrate synthase to produce citrate. The second step includes aconitase production of isocitrate from citrate. Isocitrate dehydrogenase then generates α-ketoglutarate, which is converted by α-ketoglutarate dehydrogenase into succinyl-CoA followed by succinate, which generates ATP. In the presence of fumarate dehydrogenase, fumarate is produced from succinate, which is then converted into malate by fumarase. Malate is then converted back into oxaloacetate by malate dehydrogenase. **(B)** Nuclear functions. Acetyl-CoA is used as a substrate to acetylate histones. Itaconate can inhibit TET demethylation; therefore, it inhibits the transcription of pro-inflammatory genes. α-ketoglutarate binds and activates JMJD, KDM, and TET demethylases, allowing for DNA or histone demethylation and pro- or anti-inflammatory gene upregulation. Also, it activates PHDs and therefore, supressing NFκB regulated gene transcription. Succinyl-CoA is a substrate for histone succinylation in the nucleus. Succinate can inhibit JMJD, KMDs and TET demethylation. In addition, it can inhibit KMD4A and KMD4B from demethylating H3K9 and subsequently inhibit DNA DSB repair. Fumarate inhibits KMDs TET and PHDs. Specifically, it inhibits KMD2B which allows for accumulation of H3K36me2; a key signal for DNA DSB repair

The TCA cycle takes place in the cell’s mitochondria and is used for ATP production, as shown in Fig. 1A. During this entire process, 6 nicotinamide adenine dinucleotide (NADH) molecules are combined with 1 flavin adenine dinucleotide (FADH2) and 1 ATP molecule [10]. These molecules, produced in the TCA cycle, are key components of the electron transport chain (ETC) which produces ATP for the cell by oxidative phosphorylation via ATP synthase [11, 12].

During an inflammatory response, changes also occur in the nucleus. Chromatin modifications govern the expression of multiple genes required for the immune and inflammatory response [13]. These changes include a series of histone modifications including methylation, acetylation, phosphorylation, and succinylation, to alter the DNA wrapped around the histone. Histones are positively charged proteins that package and organize DNA into chromatin, regulating gene expression through post-translational modifications. Histones are made up of 2 copies of 4 subunits: H2A, H2B, H3, and H4 [13]. DNA is covalently wrapped around histones and gene transcription is regulated by either “opening” or “closing” the DNA-histone complex, allowing it to become available or unavailable to transcription factors respectively [13]. The most common histone modifications are acetylation and methylation. Histones are acetylated by histone acetyltransferases (HATs) and deacetylated by histone deacetylases (HDACs). Histone acetylation is generally associated with active or open gene structures allowing for transcription to take place [14]. DNA methylation is carried out by an enzyme called S-adenosyl methionine (SAM) [14]. Methylation on cytosines at the 5th carbon position yielding 5-methylcytosine (5mC) is known to inhibit DNA transcription by preventing transcription factors from binding [15].

Several key metabolites and metabolic enzymes have been shown to translocate to the nucleus to influence the immune response. The earliest observation of metabolic enzymes in the nucleus was in the 1960 s when it was shown that glycolytic enzymes could translocate into the nucleus and interact with DNA and nuclear proteins [16]. In addition to performing their canonical.

Roles, metabolites and metabolic enzymes have been shown to perform non-canonical functions when translocated into the nucleus. These non-canonical or moonlighting functions refer to instances where a molecule performs tasks different to the commonly known or previously understood role. In the context of nuclear TCA cycle metabolites, this includes taking part in regulating gene transcription and participating directly or indirectly in chromatin modifications (Fig. 1B). This change in gene expression drives reprogramming of cells allowing immune cells to move from a pro-inflammatory to an anti-inflammatory state and vice versa [2]. This is a logical connection as metabolic enzymes and metabolites are sensitive to the nutrient levels in the cell and can therefore sense cellular stress [17]. This allows them to be tight regulators of gene transcription in response to changes in cellular needs.

In this review, we will be discussing the non-canonical and moonlighting functions of TCA metabolites acetyl-CoA, α -ketoglutarate, succinate, fumarate, itaconate, and succinyl-CoA in the nucleus as gene expression modulators and exploring their roles in a disease context.

### Nuclear TCA cycle metabolites

#### Acetyl-CoA

In the TCA cycle acetyl-CoA is yielded from fatty acids and amino acids, or formed from pyruvate by pyruvate dehydrogenase (PDH) when extracellular glucose is available [13, 18]. It can continue to participate in the TCA cycle by reacting with oxaloacetate to form citrate. It is distributed around the cell for various cellular needs. For example cytosolic acetyl-CoA participates in fatty acid and cholesterol synthesis by converting acetyl-CoA into malonyl-CoA: a substrate required for fatty acid synthase complex [19].

Like many other metabolites, acetyl-CoA diffuse to the nucleus passively through the nuclear pores; or is generated in the nucleus from pyruvate citrate or acetate by nuclear PDH, ATP citrate lyase (ACLY) or acyl-CoA synthetase short-chain family member 2 (ACSS2), respectively [18, 20]. Hence a role for nuclear acetyl-CoA in gene expression has become a point of interest. Acetyl-CoA is the main substrate used by HATs for histone acetylation. This means that acetyl-CoA production is directly associated with histone acetylation and regulation of gene expression: affecting cell metabolism, growth, proliferation and autophagy. Chromatin remodelling complexes such as the SWItch/Sucrose Non-Fermentable (SWI/SNF) complex rely on acetyl-CoA as part of their mechanism [21]. Therefore, a lack of lipid or glucose metabolism can alter an individual’s chromatin due to the availability of metabolites needed for chromatin modifications [22]. Acetyl-CoA derived from glycolysis is responsible for half of the acetyl-CoA used for histone acetylation. In addition, different routes of acetyl-CoA production can affect chromatin modifications and, in turn, gene expression in different ways. Production of acetyl-CoA due to high levels of acetate (a carbon donor to lipid synthesis) causes an increase in H3K9, H3K27 and H3K56 acetylation in lipid synthesis-associated genes [3, 23]. An increase of ACLY in the nucleus by Ataxia Telangiectasia Mutated (ATM) and protein kinase B (PKB)-mediated phosphorylation converts nuclear citrate to acetyl-CoA. This acetyl-CoA acts to recruit the DNA repair protein Breast cancer type 1 (BRCA1). BRCA1 is used as the substrate for the repair of double-strand breaks in DNA via homologous recombination [24]. ACSS2 is also directed into the nucleus via AMP-activated protein kinase (AMPK) phosphorylation that increases acetylation and expression at lysosomal and autophagy promoters [25].

A study by Santarsiero et al. showed how nuclear acetyl-CoA produced by ACLY contributes to the acetylation of subunit p65 of NFκB [26]. When lipopolysaccharide (LPS)-activated macrophages were treated with ACLY inhibitors they found a decrease in NFκB transcriptional activity; this included a reduction of NFκB bound to *ptgs2* and *IL-1β* gene promoters [26]. Activation of the p65 subunit by acetylation plays an important role in the cytokine storms seen in some patients infected with severe acute respiratory syndrome coronavirus 2 (SARS-CoV2) [26, 27]. Overall, the authors showed how acetyl-CoA enables crosstalk between metabolism and chromatin remodelling to induce a pro-inflammatory phenotype in macrophages during both pathogenic infection and inflammation.

#### α-Ketoglutarate

α-ketoglutarate participates in many critical metabolic pathways including glutaminolysis and the TCA cycle [28]. During glutaminolysis, the amino acid glutamine is converted into glutamate then into α-ketoglutarate by isocitrate dehydrogenase (IDH) [28]. The α-ketoglutarate then initiates and participates in the TCA cycle located in the mitochondria.

In a study by *Liu et al.*, they showed that glutaminolysis promotes anti-inflammatory macrophage polarisation due to α-ketoglutarate [29]. They showed that α-ketoglutarate translocates to the nucleus where it acts as a co-factor to a family of DNA hydroxylase called α-ketoglutarate-dependent dioxygenases. These include Jumanji C domain-containing (JMJD), lysine demethylases (KDMs) and the Ten-eleven translocation (TET). JMJD proteins use α-ketoglutarate and oxygen as co-substrates to demethylate lysine residues [30]. Therefore, when proteins in the JMJD family are activated with α-ketoglutarate they can demethylate histone sites including H3K27 on promoters of M2-specific genes. When H3K27 is trimethylated (H3K27me3), anti-inflammatory macrophage markers including *Arg1*,* YM1*,* Retnla* and *Mrc1* cannot be expressed due to the closed DNA conformation. However, in the presence of α-ketoglutarate, these genes can be expressed due to the demethylation of H3K27 and opening of the DNA conformation [29]. Concurrently, while inducing M2 macrophage polarisation, α-ketoglutarate can also repress pro-inflammatory macrophage signals [29]. α-ketoglutarate can activate prolyl hydroxylase (PHD) to hydroxylate a proline on protein kinase IKKβ; this stops the activation of IKK and inhibits NF-kB signalling [29].

In addition, α-ketoglutarate can induce TET methylcytosine dioxygenases to mark DNA for demethylation and promote gene expression. α-ketoglutarate binds to TET allowing for demethylation modification 5-hydroxymethylcytosine (5hmC) of histone-bound DNA [29]. TET-α-ketoglutarate activity has been reported to have both pro-inflammatory and anti-inflammatory consequences in macrophages depending on the stage of inflammation. TET1 has been shown to be pro-inflammatory by upregulating TNFα production during inflammation by marking its promoter with demethylation modification [31]. On the other hand, anti-inflammatory roles include TET2 regulation of the repression of inflammatory gene transcription, including IL-6 and IL-1β [32, 33]. Overall, literature has shown both a pro and anti-inflammatory response from nuclear α-ketoglutarate and its contribution to demethylase function of DNA and gene transcription.

#### Succinate

Succinate (generated from succinyl-CoA in the TCA cycle) is converted to fumarate by succinate dehydrogenase [13]. Like all of the TCA cycle metabolites its main function is to generate cellular ATP in the mitochondria [34]; however succinate has also been shown to induce a pro-inflammatory response by inhibiting PHD which in turn promotes Hif1α and increases production of IL-1β [35]. Furthermore, when the enzyme succinate dehydrogenase (SDH) is inhibited, there is an upregulation in M2 anti-inflammatory-associated genes and a decrease in M1 pro-inflammatory-related genes during cytosolic localization [36].

Succinate can be translocated into the nucleus by passive diffusion through nuclear pores or produced in the nucleus from nuclear α-ketoglutarate. However, when nuclear SDH is mutated or downregulated, this can cause succinate accumulation. In a study by Xiao et al., the effect of succinate on global histone methylation was investigated [30]. Knock down of SDH lead to a reduction in fumarate, an increase in succinate and a significant increase in methylation of H3K4, H3K9 and H3K79, suggesting that succinate competitively inhibits α-ketoglutarate-dependent dioxygenases including TET, KDMs and JMJDs [30]. Succinate accumulation also impacted TET1 and TET2 activity. In SDH knockdown cells, there was a significant decrease in TET1-1 and TET2-induced 5hmC due to succinate accumulation [30]. Another study showed that succinate accumulation could influence gene expression and cause tumorigenesis [30]. High levels of succinate could reduce homologous recombination by suppressing two particular lysine demethylases, KDM4A and KDM4B. This caused DNA double-strand breaks to increase using a pathway mediated by KDM4A and KDM4B [37]. Overall, succinate accumulation could be considered an oncogenic mechanism, as it causes an increase of double-strand breaks in DNA by inhibiting demethylase activity resulting in tumour growth.

#### Fumarate

Fumarate is a TCA cycle metabolite produced in the mitochondria and nucleus by fumarate hydratase (FH). Inactivating mutations or inhibition of FH have been shown to promote an inflammatory macrophage phenotype by decreasing IL-10 and upregulating TNFα with LPS stimulation [38]. In addition, the loss of FH activity leads to fumarate accumulation, promoting ‘succination-by-fumarate’; a spontaneous reaction of fumarate with thiol cysteines producing (succino)cysteine (2SC) [39]. This has shown to occur in the mitochondria, cytosol and nucleus. In the nucleus, fumarate can use this modification to inhibit SMARCC1, a core subunit in the SWI/SNF complex. This leads to a decrease in SNF5 tumour suppressor which is linked to promotion of hereditary leiomyomatosis and renal cell carcinoma (HLRCC) [40, 41].

Like succinate, nuclear fumarate is known to increase methylation by competitively inhibiting α-ketoglutarate-dependent dioxygenases including KDMs, TET1 and TET2 and PHDs [30]. In addition, nuclear fumarate also promotes DNA double-strand break repairs caused by inflammation [42]; when DNA damage is sensed, FH translocates to the nucleus to the site of the DNA double-strand breaks (DSB) [43]. At the DSB, the DNA becomes open for repair in the presence of H2A.Z histone variants (part of the histone H2 family) [43]. These histone variants allow for a more dynamic nucleosome as they have a less positively charged C-terminus tail which causes a reduction of interactions with the DNA and easier unwinding of DNA [44, 45]. The relaxed DNA structure induces the binding of the BRCA1 complex and Ku70/Ku80 proteins for the non-homologous end-joining (NHEJ) pathway for DNA repair [45]. H3K36me2 recruits DNA repair proteins, including p53-binding protein 1 (53BP1) to the DSB [46]. When FH translocates to the nucleus, it is phosphorylated by DNA-PK on Thr 236. This leads to the binding of FH to H2A.Z at DNA DSB [43]. Bound FH produces a local source of fumarate in the nucleus, which has been found to inhibit the demethylase KDM2B [47]. Fumarate has a similar structure to α-ketoglutarate which allows fumarate to bind to α-ketoglutarate-dependent demethylases and competitively inhibit them. KDM2B histone demethylase reduces H3K36me2 but is inhibited in the presence of fumarate. An increase in H3K36me2 recruits more DNA repair proteins to the site of DSB [43]. This exhibits an anti-inflammatory effect of nuclear fumarate by aiding in DNA repair, thereby promoting cell survival and possibly anti-cancer properties.

#### Itaconate

Itaconate has become a highly researched metabolite and is found to have anti-inflammatory, anti-bacterial and anti-viral properties. Itaconate’s anti-inflammatory nature is in part due to its ability to post-transcriptionally modify cysteines on regulatory proteins via ‘Michael’s addition-based mechanism’ [48]. This modification produces a stable thioester bond which can alter structures, protein interactions and enzymatic functions [49]. In addition, itaconate also regulates inflammation by acting as an enzyme inhibitor. In a recent study carried out by *Chen et al.*, itaconate was discovered to bind to TET2, suggesting nuclear localization of the metabolite [50]. Immune-responsive gene 1 (IRG1) is the enzyme responsible for catalysing itaconate production. HEK293T cells expressing either wild-type *Irg1* or a catalytically inactive *Irg1* mutant were used to investigate the impact of itaconate accumulation on global histone and DNA demethylation. The results showed that itaconate can competitively inhibit nuclear TET2 enzymes thus inhibiting certain chromatin modifications [50]. TET2 is recruited by NFκβ during inflammatory conditions. Once recruited, TET2 oxidises methylated cytosine in DNA to 5-hmC allowing regulation of gene expression by DNA methylation. As a resolution regulator of inflammation, IRG1 is upregulated in early stages of inflammation and subsequently, itaconate levels increase[50]. Itaconate can inhibit TET2 not only due to its structural similarity to α-ketoglutarate, a known TET2 substrate, but also because of its spatial proximity. Both itaconate and α-ketoglutarate have a dicarboxylic acid with 4–5 carboxylate groups. Itaconate competitively binds to the TET2 catalytic site, preventing α-ketoglutarate binding and inhibiting TET2. Confirming itaconate’s role as a TET2 inhibitor, *Irg1*-deficient cells showed a decrease in 5-hmC in the DNA compared to WT cells, indicating that the TET2 enzyme was inhibited by itaconate. WT cells also showed downregulation of TET2-induced inflammatory genes while *Tet2*-KO cells showed little effect on gene expression when treated with itaconate. This reveals that TET2 and itaconate modulate the same LPS-induced genes suggesting they cooperate [50]. This link between TET2 and itaconate can help explain itaconate’s anti-inflammatory effects on LPS-induced cells. It would be interesting to see how this mechanism works in disease pathogenesis in future research.

#### Succinyl-CoA

The α-ketoglutarate dehydrogenase complex (α-KGDH) is also present in the nucleus and can produce succinyl-CoA from α-ketoglutarate. Nuclear succinyl-CoA plays a role in posttranslational modifications such as succinylation, whereby a succinyl group is added to lysine residues of proteins, altering their structure, function, and interactions [34]. It has been found that enzymatically mediated succinylation is almost entirely contingent on succinyl-CoA availability in the nucleus. When the α-KGDH is translocated into the nucleus it binds to lysine acyltransferase 2 A (KAT2A), which allows local production of succinyl-CoA. KAT2A is a histone acetyltransferase and a succinylase that binds acetyl-CoA but competitively favours succinyl-CoA due to its higher affinity [51]. Lysine succinylation causes significant changes to protein structure due to the bulky nature of the succinyl group [52]. Lysine succinylation is important for conformational changes and protein-protein interactions due to the involvement of the succinyl chain in non-covalent interactions including van der Waals interactions, hydrogen bonds, and electrostatic binding with negatively charged residues [52]. Histone succinylation, facilitated by succinyl-CoA, has been observed at transcriptional promoters and is linked to the regulation of gene expression [53].

Loss of function of SDH in the nucleus leads to the accumulation of succinyl-CoA resulting in hyper-succinylation of histones proving that succinylation is dependent on local succinyl-CoA availability [54]. In a study by Liu et al., they found that H3K79 succinylation occurred in promoter regions of *IK3R1*, *JUN* and *PRKDC.* Researchers demonstrated that α-KGDH-coupled KAT2A can promote tumour growth. To show this, they intracranially injected athymic nude mice with KAT2A-deficient U87 reconstituted with flag-tagged KAT2A mutants. This showed a decrease in *IK3R1*, *JUN* and *PRKDC* gene expression and inhibition of brain tumour growth [51]. Not only does this show the importance of nuclear α-KGDH in tumour growth, but demonstrates the functional consequence of nuclear succinyl-CoA and succinylation.

### Moonlighting nuclear functions of metabolic enzymes

So far, this review has focused on the canonical role these metabolic enzymes play in the nucleus. There is ample evidence that TCA cycle enzymes translocate to the nucleus, as previously described α-ketoglutarate dehydrogenase, succinate dehydrogenase and FH are shuttled into the nucleus to alter the nuclear metabolite pool with functional consequences. However, there is also evidence that many metabolic enzymes can perform secondary and non-canonical functions in the nucleus via both enzymatic and non-enzymatic mechanisms utilising a process known as moonlighting. Whilst research into moonlighting functionality is limited, new data is emerging that suggests several enzymes may be operating in a moonlighting capacity [53].

Pyruvate kinase muscle isozyme 2 (PKM2) is a glycolytic enzyme responsible for catalysing the last step of glycolysis, converting phosphoenolpyruvate (PEP) to pyruvate. Beyond the role in glycolysis, dimeric PKM2 can translocate into the nucleus to modulate gene expression via histone modifications [55–57]. Nuclear translocation is initiated by extracellular signal-regulated kinase (ERK) phosphorylating Ser37 on PKM2. This allows for peptidyl-prolyl cis–trans isomerase NIMA-interacting 1 (PIN1) binding. PIN1 facilitated the interaction between PKM2 and importin α5, allowing nuclear translocation. When in the nucleus, PMK2 acts as a co-activator for hypoxia-inducible factor 1-alpha (HIF-1α). PHD3 acts as a cofactor of PKM2 which allows for HIF-1α transactivation of glycolic enzymes, including lactate dehydrogenase (LDH), the glucose transporter GLUT-1, and pyruvate dehydrogenase kinase-1 (PDK-1) [58–60]. This is an important mechanism of HIF-1α as a key modulator in the Warburg effect by driving. Induction of these enzymes allows for the maintenance of aerobic glycolysis [58]. When PMK2 is bound to β-catenin, it is recruited to the nucleosome where it phosphorylates threonine 11 on H3 thereby promoting H3 acetylation [57]. This modification has been shown to regulate EGFR-mediated gene expression, cell proliferation and transcription [61]. Recently, it has been found that nuclear translocation of PKM2 can lead to increased aerobic glycolysis in cancer-associated fibroblasts (CAFs). Therefore, by blocking PKM2 nuclear translocation, researchers can stop circABCC4-driven oxaliplatin resistance of pancreatic cancer in vivo [62, 63].

Glyceraldehyde-3-phosphate dehydrogenase (GAPDH) is another glycolytic enzyme that has moonlighting functions in the nucleus [64]. GAPDH translocates to the nucleus upon cellular stress complexed with seven in absentia homolog (SIAH) [65, 66]. Once in the nucleus, GAPDH is acetylated by p300/CBP, known HATs, that in turn activates the catalytic activity of p300/CBP to target p53 tumour suppressor pathway activation [67, 68]. GAPDH is also able to affect DNA repair, DNA transcription, and cell cycle regulation by inducing gene expression of uracil DNA glycosylase (UDG), apurinic/apyrimidinic endonuclease (APE1), octamer transcription factor 1 (OCT-1) and SET nuclear proto-oncogene (SET), respectively [65]. The outcomes of nuclear GAPDH are aligned with the anti-inflammatory phenotype as it functions to combat inflammation and cellular stress by promoting gene expression of DNA repair and cell cycle-regulating proteins.

Based on the nuclear roles of several glycolytic enzymes, further investigation into the TCA cycle enzyme’s non-canonical functions in the nucleus is warranted.

### Clinical and disease context

#### Inflammatory disease

DNA methylation and histone acetylation are key modifications that play a crucial role in gene regulation. Their dysregulation has been implicated in the development of various inflammatory disorders (Table 1) [54]. These changes are found to be a direct consequence of metabolic changes and metabolite availability [69]. As previously explained, many mitochondrial metabolites and metabolic enzymes play a key role in gene modulation. However, the availability of these metabolites is dependent on inflammatory responses driving metabolic reprogramming [70].

Sepsis is an inflammatory disorder where the relationship between TCA cycle metabolites and chromatin modifications can influence disease pathology [71]. During inflammation, downregulation of TCA cycle enzyme activity contributes to an accumulation of metabolites such as succinate [71]. Increased expression of *Irg1*, leads to an upregulation of itaconate, which in turn inhibits SDH, promoting the accumulation of succinate [72]. When succinate accumulates, the α-ketoglutarate/succinate ratio is decreased resulting in inhibition of α-ketoglutarate-dependent dioxygenases like TET and JMJDs [30]. As previously mentioned, itaconate can inhibit TET2 and has been shown to decrease inflammatory gene expression [50]. In one study, Shen et al., showed that TET2-deficient mice were better protected from sepsis than WT mice. TET2 promotion of sepsis has been shown to occur due to TET2 suppression of Soc3 expression. Soc3 is a negative regulator of the pro-inflammatory JAK-STAT pathway and suppressor of myelopoiesis induction [73]. Therefore, succinate and itaconate could contribute to the reduction of sepsis by regulating TET2.

**Table 1:** Non-canonical nuclear functions of TCA cycle metabolite and disease progression as a functional consequence

Metabolite	Nuclear function	Disease	Cell type	Reference
Acetyl-CoA	• Substrate for histone acetylation to regulate gene expression • Recruit BRCA1 to resolve DNA DSB • Acetylation of p65 on NFκB to downregulate *PTAG2* and *IL-1β*	n/a	• HCT116 colorectal cancer cell lines • prototrophic CEN.PK strain of Saccharomyces cerevisiae • Primary human monocytes and iBMDMs	[23, 24, 26]
α-ketoglutarate	• Acts as a cofactor for α-ketoglutarate dependent dioxygenases including TET, JMJD and KDMs • Activate PHDs to hydroxylate a proline on IKKβ	n/a	• HEK293T cells • OT-1 CD8 + T cells and BMDMs	[29, 30]
Succinate	• Inhibits α-ketoglutarate dependent dioxygenases including TET1-2 and JMJD • Supress KDM4A and KMD4B to reduce DNA DSB repair	• Increase in SDH PGL/PCC	• HEK293T cells • cortical renal YUNK1 cells from a patient with kidney cancer	[30, 37]
Fumarate	• Succination of thiol cysteines to 2SC • inhibiting α-ketoglutarate-dependent dioxygenases including TET1 and TET2 and PHDs • Inhibits KMD2B to increase DNA DSB repair	• Increase risk of HLRCC	• Macrophages • T-cells • HEK-293 cells • U2OS cells	[39–41, 43]
Itaconate	• Inhibits TET2 to reduce inflammatory gene transcription	• Decrease risk of sepsis • Decrease bone destruction by rheumatoid arthritis	• THP1 derived macrophages • BMDMs and PBMCs	[50, 74]
Succinyl-CoA	• Succinylation post-modification to alter protein structure • Succinylation of histones	• Promotes tumorigenesis	• Parental U87, U251, and 293 cells	[51, 54]

In a recent study, Rong et al., identified that itaconate is produced by macrophages in bone tissue during the development of rheumatoid arthritis (RA) [74]. RA is a well-known autoimmune disorder causing chronic inflammation of joints. In many cases, RA can lead to bone destruction and deformities of joints. They were able to identify itaconate as a key metabolite in RA by performing a metabolome analysis on the synovial fluid of RA patients. They also produced *Irg1* ^*−*^*/*^*−*^/TNF-Tg mice to determine if the lack of itaconate would drive RA faster than *Irg1* ^*+*^*/*^*+*^/TNF-Tg mice. They concluded that a deficiency of IRG1 causes faster RA development and more severe bone destruction. After performing RNA-seq, they found that similar genes were downregulated in itaconate-treated and TET-deficient samples. Of these genes, Acp5, Ctsk, and Mmp9 are known to play a role in RA pathogenesis. These genes were downregulated when treated with OI (an itaconate derivative), showing itaconate can inhibit RA progression and bone destruction by reducing osteoclast differentiation by inhibiting TET2 [74]. This study shows potential for itaconate-derived therapeutics for RA and bone destruction disorders.

Other inflammatory diseases driven by epigenetic dysregulation are Alzheimer’s, psoriasis, asthma, and inflammatory bowel disease [75]. A potential link between these diseases and the function of nuclear metabolites may exist due to the heavy influence of metabolite regulation on epigenetics in the context of inflammation via mechanisms such as DNA methylation and histone modification. Succinate accumulation is also associated with obesity, diabetes mellitus, renal complications, and cardiovascular disease, and while not directly linked to nuclear succinate accumulation, it could be an unexplored contributing factor [71]. 

Our understanding of the precise mechanisms underlying these interactions remain incomplete. Further research is needed to elucidate how these metabolic changes affect gene expression and immune responses, which could provide new insights into therapeutic strategies for inflammatory disorders.

#### Oncometabolites

Research indicates that mutations in metabolic enzymes can contribute to cancer development by leading to the accumulation of oncometabolites, which disrupt cellular signalling and epigenetic regulation. This aberrant accumulation of metabolites can disrupt egene modulation when they translocate to the nucleus. Loss of FH and SDH has been specifically found to drive cancer progression through an accumulation of nuclear fumarate and succinate, leading to inhibition of demethylation enzymes in the nucleus [76, 77]. In addition, mutations in FH also inhibit DNA repair mechanisms driving tumorigenesis by genomic instability [78]. SDH mutations have been linked to hereditary paraganglioma (PGL) and pheochromocytoma (PCC). The accumulation of succinate can promote tumorigenesis by again decreasing demethylation of DNA [79]. In addition, mutations in FH have been specifically linked to HLRCC caused by DNA hypermethylation due to TET inhibition. Consequently, this drives the epithelial-mesenchymal transition (EMT) which is a common characteristic of metastatic tumours [80]. EMT is a common process in the initiation, invasion and metastasis in cancer progression. Succinylation by the α-KGDH complex, using succinyl-CoA, promotes tumorigenesis and prevents α-KGDH from entering the nucleus, attenuating tumour growth [51]. Overall, accumulation of TCA metabolites in the nucleus can lead to devastating disease and a potential area for cancer therapeutics. 

## Conclusion

Overall, TCA cycle metabolites have been shown to regulate inflammation by modulating epigenetics and gene expression. Further research is required to elucidate the non-canonical nuclear functions of TCA cycle enzymes. While existing literature explores the connection between the TCA cycle and inflammatory diseases, further investigation into gene expression modulator roles of these metabolites in disease pathogenesis will no doubt reveal nuclear metabolites to be key regulators of gene expression.

## Supplementary Information

Supplementary Material 1.

## Data Availability

No datasets were generated or analysed during the current study.

## Abbreviations

TCA: Tricarboxylic acid cycle
ATP: Adenosine triphosphate
NADH: Nicotinamide adenine dinucleotide
FADH2: Flavin adenine dinucleotide
ETC: Electron transport chain
DNA: Deoxyribose nucleic acids
HATs: Histone acetyltransferases
HDACs: Histone deacetylases
SAM: S-adenosyl methionine
5mC: 5-methylcytosine
PDH: Pyruvate dehydrogenase
ACLY: ATP citrate lyase
ACSS2: Acyl-CoA synthetase short-chain family member 2
SWI/SNF: SWItch/Sucrose Non-Fermentable
ATM: Ataxia Telangiectasia Mutated
PKB: Protien kinase B
BRCA1: Breast cancer type 1
AMPK: AMP-activated protein kinase
LPS: Lipopolysaccharide
SARS-CoV2: Severe acute respiratory syndrome coronavirus 2
IDH: Isocitrate dehydrogenase
JMJD: Jumanji C domain-containing
KMDs: Lysine demethylases
TET: Ten-eleven translocation
PHD: Prolyl hydroxylase
5hmC: 5-hydroxymethylcytosine
SDH: Succinate dehydrogenase
FH: Fumarate hydratase
2SC: (succino)cysteine
HLRCC: Hereditary leiomyomatosis and renal cell carcinoma
DSB: DNA double-strand breaks
NHEJ: Non-homologous end-joining
53BP1: p53-binding protein 1
IRG1: Immune-responsive gene 1
α-KGDH: α-ketoglutarate dehydrogenase complex
KAT2A: Lysine acyltransferase 2 A
PKM2: Pyruvate kinase muscle isozyme 2
PEP: Phosphoenolpyruvate
ERK: Extracellular signal-regulated kinase
PIN1: Peptidyl-prolyl cis–trans isomerase NIMA-interacting 1
HIF-1: Hypoxia-inducible factor 1-alpha
LDH: Lactate dehydrogenase
PDK-1: Pyruvate dehydrogenase kinase-1
CAFs: Cancer-associated fibroblasts
GAPDH: Glyceraldehyde-3-phosphate dehydrogenase
SIAH: Seven in absentia homolog
UDG: Uracil DNA glycosylase
APE1: Apurinic/apyrimidinic endonuclease
OCT-1: Octamer transcription factor 1
SET: SET nuclear proto-oncogene
RA: Rheumatoid arthritis
PGL: Paraganglioma
PCC: Pheochromocytoma
EMT: Epithelial-mesenchymal transition
